# Approaches of Inducing Tolerance to Murine *Schistosomiasis mansoni* applying *Biomphalaria* and *Bulinus* Proteins

**DOI:** 10.1007/s11686-025-00988-2

**Published:** 2025-01-24

**Authors:** Hanan S. Mossalem, Sami Mohamed Nasr, Azza Moustafa Fahmy, Shimaa Atta, Gehan Labib Abuelenain

**Affiliations:** 1 Medical Malacology Department, Theodor Bilharz Research Institute, Giza, 12411 Egypt; 2 Biochemistry and Molecular Biology Department, Theodor Bilharz Research Institute, Giza, 12411 Egypt; 3 School of Biotechnology, Badr University in Cairo, Badr City, Cairo 11829 Egypt; 4 Parasitology Department, Theodor Bilharz Research Institute, Giza, 12411 Egypt; 5 Immunology Department, Theodor Bilharz Research Institute, Giza, 12411 Egypt

**Keywords:** Schistosomiasis, *Shistosoma mansoni*, *Schistosoma haematobium*, Snail proteins, Nucleoprotein, *Bulinus truncatuse*, *Biomphalaria alexandrina*

## Abstract

**Background:**

The freshwater snails *Biomphalaria alexandrina* and *Bulinus trancatus* are key contributors to the transmission of *S. mansoni* and *S.haematobium*, respectively, for being their intermediate hosts.

**Objectives:**

This research study aimed to investigate the potency of the nucleoproteins (NPs) extracted from both snail species on the host immune reactions as an approach to developing a potential vaccine.

**Methods:**

Three groups of six-week-old Swiss-Webster mice (*n* = 18; 15–20 g each) were injected intraperitoneally for three consecutive weeks with single doses (once a week) of *B. alexandrina*, *B. truncatus*, or a mixture of their nucleoproteins (50 µg each). On day 21st, the nucleoprotein-treated mice altogether, with six more mice, received subcutaneously *S. mansoni* cercariae (60/mouse). Eight weeks later, the experimental mice were sacrificed for evaluation of certain parasitological, molecular and immunological responses.

**Results:**

The data of mice immunized with the various types of nucleoproteins showed a significant increase of FAS/R gene expressions in hepatic tissues and anti-IgG antibody levels in sera on the one hand and a significant decrease of worm loads and β-actin/R gene expression levels on the other hand when compared to the infected control mice.

**Conclusion:**

These findings highlight the role of snails in immunomodulation and shed light on the possibility of antagonizing effects that might occur when the nucleoproteins of different species are mixed. Moreover, this research study might promote the literature spotting the importance of snail proteins against schistosomiasis.

**Supplementary Information:**

The online version contains supplementary material available at 10.1007/s11686-025-00988-2.

## Introduction

Schistosomiasis distresses up to 600 million people in 74 tropical and sub-tropical countries, mostly in the developing world [8]. The disease is caused by several *Schistosoma* species that might induce either urinary or hepatic severe implications (WHO, 2023). Among the different schistosome species, *Schistosoma mansoni* is the most abundant in Egypt [9] and one of the prominent eco-society and health challenges in developing countries. Chemotherapeutics are sufficient enough to kill the adult *Schistosoma* parasite, yet they are poor agents against immature schistosome stages [17] and unreasonable for the prevention of the re-emerged disease [34]. Moreover, there is a demonstrated emergence of *S. mansoni* resistance to praziquantel. Therefore, there is urgency for new approaches to control and prevent schistosomiasis [31]. Schistosomiasis control measures focus on breaking the parasite lifecycle. These measures Mass drug administration (MDA) with praziquantel to eradicate the inhabitant worms in infected individuals. Vector control, such as environmental management and mollusciciding, reduces the population of snail intermediate hosts. Health education promotes awareness about avoiding risky behaviors and maintaining hygiene. Community-based interventions and surveillance programs help sustain progress and monitor re-emergence. Integrated approaches combining these strategies ensure long-term disease control and prevention [1, 2].

Mollusca NPsare well established as a source of bioactive components and immunostimulants to control schistosomiasis. The snail mucus was proven to be antimicrobial agent and have repair and regeneration effect for the liver of *S. mansoni-*infected *mice* [14, 15]. For human as wound healing agent, chronic bronchitis therapy [35], Snails are well known to play an important role in homeostasis and protection of the gastric mucosa as well, and their nucleotides might have a therapeutic effect on human liver cirrhosis [12].

Mucus and NPs had a prophylactic effect as antischistosomal therapeutics regarding their cercaricidal, miracidicidal and anti-schistosomal effect in vitro. This can be applied by using formulations containing treatments of B. alexandrina mucus or NPs in water canals to benefit from its cicaricidal and miracidicidal effects as a biological control measure. Moreover, their combination could be considered as a sharing therapeutic adding to PZQ potentiality in vivo for further investigations [28]. A recent study has suggested that snails can manipulate the *Schistosoma haematobium* immune response to defend themselves [20].

Therefore, the current study aims to investigate the effect of the protein produced from the intermediate host *B. alexandrina* and *B. truncatus* on the immune system of an experimental definitive host, mice.

## Materials and Methods

### Snail Breeding

Egg masses (*n* = 100) were collected from *Biomphalaria alexandrina* and *Bulinus truncatus* snails breeding in the Medical Malacology lab at Theodor Bilharz Research Institute. In individual divided plates, every 50 eggs were incubated with distilled water (10 ml) and algae at 25 ± 1 °C till they reached 3 mm shell diameter/shell height. Then, baby snails were transferred to an aquarium and fed on dried lettuce leaves to attain the 5–8 mm shell diameter/shell height [10].

### Snail Tissues

Calcareous-shell-free, soft tissues were extracted and collected from mature, healthy snails by crushing them between two glass slides. Then, the tissue was stored at -20^o^C for molecular processing.

### Schistosoma Mansoni Cercariae Shedding

*B. alexandrina* snails (2–4 mm shell diameter) were infected by miracidia form Schistosome Biological Supply Centre at Theodor Bilharz Research Institute “SBSC” (5–8 each) applying a stereomicroscope. The snails were exposed to light for a couple of hours, followed by keeping them in the dark for 21–30 days at 25 ± 1 °C. Food and examination of cercariae shedding were implemented during that period [14].

### Nucleoprotein Extraction and Quantification

A series of buffers were applied to extract the nucleoproteins from the soft tissues of *S. mansoni*-infected and non-infected *B. alexandrina* and *B. truncatus* as described [18]. Tissue was first processed by buffer A [PBS (pH 7.4), NaCl (137 mM), KCl (2.7 mM), Na_2_HPO_4_ (10 mM), KH_2_PO_4_ (2 mM)], followed by buffer B [HEPES (pH 7.9, 20 mM), MgCI_2_ (1.5 mM), 25% glycerol, NaCl (420 mM), EDTA (0.2 mM), DTT (1 mM)], and finally treated with a recovery buffer C [HEPES (pH 7.9, 20 mM), 20% glycerol, KCI (100 mM), EDTA (0.2 mM), DTT (1 mM)]. According to [29], bovine serum albumin; BSA (0.1gm) was dissolved in GdnHCl (10 ml, 1 M) to achieve a final protein concentration of 1 mg/ml. Serial dilutions were performed as shown in Table 1.

**Table 1:** Bradford BSA serial dilutions

Concentration	µl from (1 mg/ml)	µl of dH_2_O	GdmClCL Absorbance (1 M)	NaCl Absorbance (0.1 M)
50 µg/ml	25 µl	475 µl	1.02	1.075
100 µg/ml	50 µl	450 µl	1.608	1.62
200 µg/ml	100 µl	350 µl	1.981	1.986
300 µg/ml	150 µl	400 µl	1.575	1.903
400 µg/ml	200 µl	375ul	1.913	1.992
500 µg/ml	250 µl	250 µl	1.952	1.966
600 µg/ml	300 µl	200 µl	1.938	1.997
700 µg/ml	350 µl	150 µl	1.957	1.981
800 µg/ml	400 µl	100 µl	1.966	1.992
1000 µg/ml	500 µl	-------	1.977	1.995

To detect the concentration of nucleoproteins, 30 µl from the extract was mixed with 470 µl of Bradford (BioRad) and measured at 595 nm. The obtained OD at 595 nm was 1; samples were diluted before the measurement to 1:10. Thus, the OD at 595 was 20. The curve of GdnCl was applied to calculate the concentration of each sample, from the curve calibration; it was revealed that the final extracted nucleoproteins concentration in HEPES buffer was (500 µg/ml).

### Experimental Design

Six-week-old female Swiss-Webster mice (15–20 g) were maintained in the animal house of Theodor Bilharz Research Institute (TBRI, Giza, Egypt) according to the guidelines of the National Institutes of Health (NIH) for animal care. A total of 24 mice were injected subcutaneously with *S. mansoni* cercariae (60 each) on day zero. The infection was preceded by three single doses (50 µg each) of nucleoproteins, administered intraperitoneally once a week over three consecutive weeks. Mice were deployed into four groups, including infected mice controls, infected and pre-treated mice with either *B. alexandrina* nucleoprotein, *B. truncatus* nucleoprotein, or mixed nucleoproteins of both snail species. Mice were sacrificed 8 weeks post-infection for estimation of parasitic, molecular, and immunologic response.

### Antigen Preparation and ELISA

Soluble Egg Antigen (SEA) was prepared and characterised by the technique already established in our lab [25]. One million eggs were homogenised using a manual homogeniser for at least 20 min according to [16]. The homogenate was centrifuged for 2 h using a high-speed ((8500x) cooling (4 °C) Eppendorf centrifuge. After centrifugation, the lipid was removed from the top of the homogenates using a tiny filter paper. The supernatant was then centrifuged for one hour using an ultracentrifuge (4 °C; 33000x). The supernatant was then aliquoted and frozen (-20 °C) for at least 2 h. The aliquots were lyophilised overnight using an Eppendorf lyophiliser. The protein content of the sample was measured according to Stratford (1969). The homemade semiquantitative ELISA was performed to determine IgG in serum samples of mice in different test groups. The plates were coated with 100 µl/well of SEA (30 µg/ml) overnight at 4 °C and blocked using 100 µl/well of carbonate buffer. The optimal dilution of serum was 1:100, and samples were incubated overnight at 4 °C. Anti-mouse polyvalent antibody-labelled with horse radish peroxidase (sigma, Germany) at a concentration of 1:1000 and OPD were used to visualize the attached Abs. Optical density was estimated at 492 nm.

### Total RNA Extraction

Homogenize liver tissue samples of 0.2 gm in 1 ml of TRIZOL reagent (TRIzol™ Reagent Catalog #: 15596026, ThermoFisher) per 100 mg of tissue. 200 µl of chloroform were added per 1 ml of TRIZOL Reagent and Vortexed vigorously for 15 s and incubated at room temperature for 2 to 3 min [5]. Mixture were Centrifuged at 12,000 x g for 15 min at 40 C. Following centrifugation, the mixture separated into lower red, phenol-chloroform phase, an interphase, and a colorless upper aqueous phase. RNA remains exclusively in the aqueous phase. The upper aqueous phase was carefully transferred without disturbing the interphase into a fresh tube. RNA was precipitated from the aqueous phase by mixing it with 0.5 ml of isopropyl alcohol. Samples were incubated at room temperature for 10 min and centrifuged at 12,000 x g for 10 min at 4^o^C [4]. RNA pellet was washed once with 1 ml of 75% ethanol and centrifuged at 7,500 x g for 5 min at 4^o^C twice. RNA was dried for 30 min at room temperature. Then, RNA was dissolved in 50 µl of DEPC-treated water [15]. The RNA concentration and purity were confirmed using the relative absorbance ratio at 260/280 on a nanodrop 2000 (Thermo, Wilmington, USA). RNA samples with a ratio higher than 1.8 were used for RT-qPCR [37].

### Reverse Transcription

Reverse transcription according to the manufacturer’s protocol RevertAid First Strand cDNA Synthesis kit (ThermoFisher Scientific, USA). A total RNA (2µL) was mixed with Oligo (dT)18 primer (1µL), reaction Buffer (4µL; 5X), RiboLock RNase Inhibitor (1µL; 20 U/µL), dNTP mix (2µL; 10mM) and RevertAid M-MuLV RT (1µL; 200 U/µL). Reverse transcription was performed at 52 °C for 60 min, followed by an inactivation reaction at 70 °C for 5 min. The resulting cDNA was stored at -20^o^C until use [36].

### Molecular Estimation for FAS and β-Actin Genes

Primer design for FAS receptor and β-Actin genes: Primers were designed using the online server called primer3 (http://bioinfo.ut.ee/primer3-0.4.0/) with specific conditions to give high quality, clear amplification and regarding the validation of the designed primer, done by the help of another online server IDT Oligo Analyzer (https://www.idtdna.Com/Primer Quest) for checking the validity and the integrity of the primers and if they have any hairpin form or hetero/homo primer dimer, which revealed clear and absolute integrity for the structure and validity. The FAS-F primer was 5-GTTTTCCCTTGCTGCAGACA-3, while the FASR primer was 5-TTGACAGCAAAATGGGCCTC-3, β-Actin-F primer was 5-GGGAATGGGTCAGAAGGACT-3 and the β-Actin-R primer was 5-CTTCTCCATGTCGTCCCAGT-3. A real-time PCR proceeded as follows: cDNA (5µL), SYBR Green/ROX qPCR Master Mix (12.5µL; SimplyGreen qPCR Master Mix, Rox, GeneDirex, Taiwan), forward primer (0.3 ml; 10 mM), reverse primer (0.3µL; 10 mM), and nuclease-free water were mixed to make a 25 µL reaction volume. The reaction was done twice by increasing the concentration of the cDNA. The PCR technique was performed using a two-step process: 95 °C for 10 min, 50 cycles at 95 °C for 15s and 57 °C for 60 s. Then, dissociation curves (DC) and melting temperatures (Tm) were recorded.

## Results

The parameters analyzed include the mean number of worms, optical density (O.D.) for anti-Schistosoma IgG, and gene expression levels of FAS and β-Actin genes. Data are represented as mean ± standard error.

### Effect of Snail-Derived Proteins on Worm Burden

**Fig. 1:**
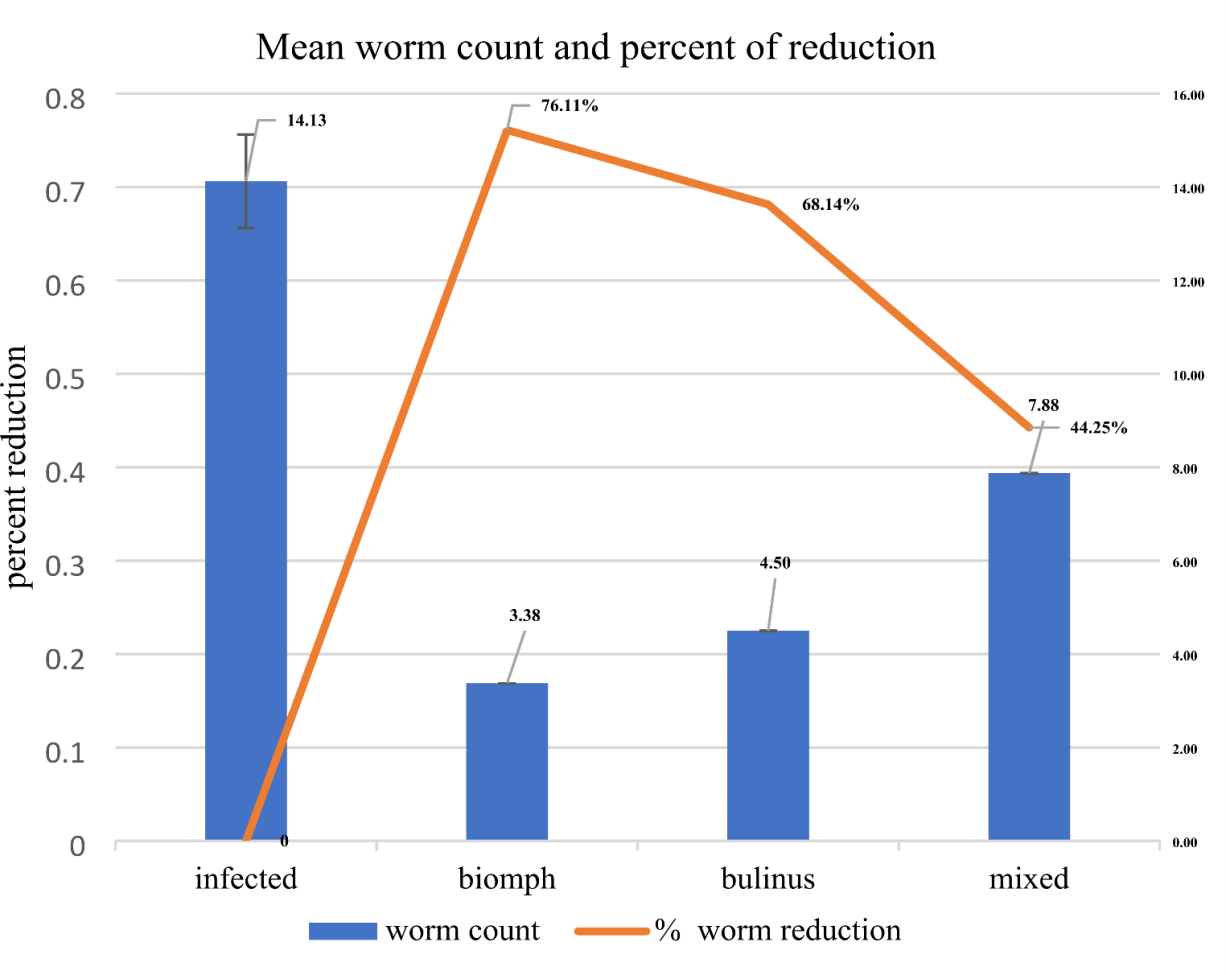
Mean worm count and reduction percentage across treatment groups. Data are represented as mean ± standard errors. **Significant difference at *p* < 0.01, NS is non significant

The comparison of worm burden among different experimental groups revealed a significant reduction in worm counts for all treatment groups compared to the infected control group. The infected control group exhibited the highest mean worm count of 14.13 ± 1.51, serving as the comparison baseline. The Biomphalaria group showed a substantial decrease in mean worm count, recording 3.38 ± 0.71 worms, which corresponds to a 76.11% reduction (*p* < 0.01) compared to the control group. Similarly, the Bulinus group demonstrated a significant reduction, with a mean worm count of 4.50 ± 1.16 and a 68.14% reduction (*p* < 0.01) relative to the control group. The Mixed Species group exhibited a mean worm count of 7.88 ± 1.84, representing a 44.25% reduction (*p* > 0.05) in worm burden (Fig. 1).

### Anti-Schistosoma IgG Levels

**Fig. 2:**
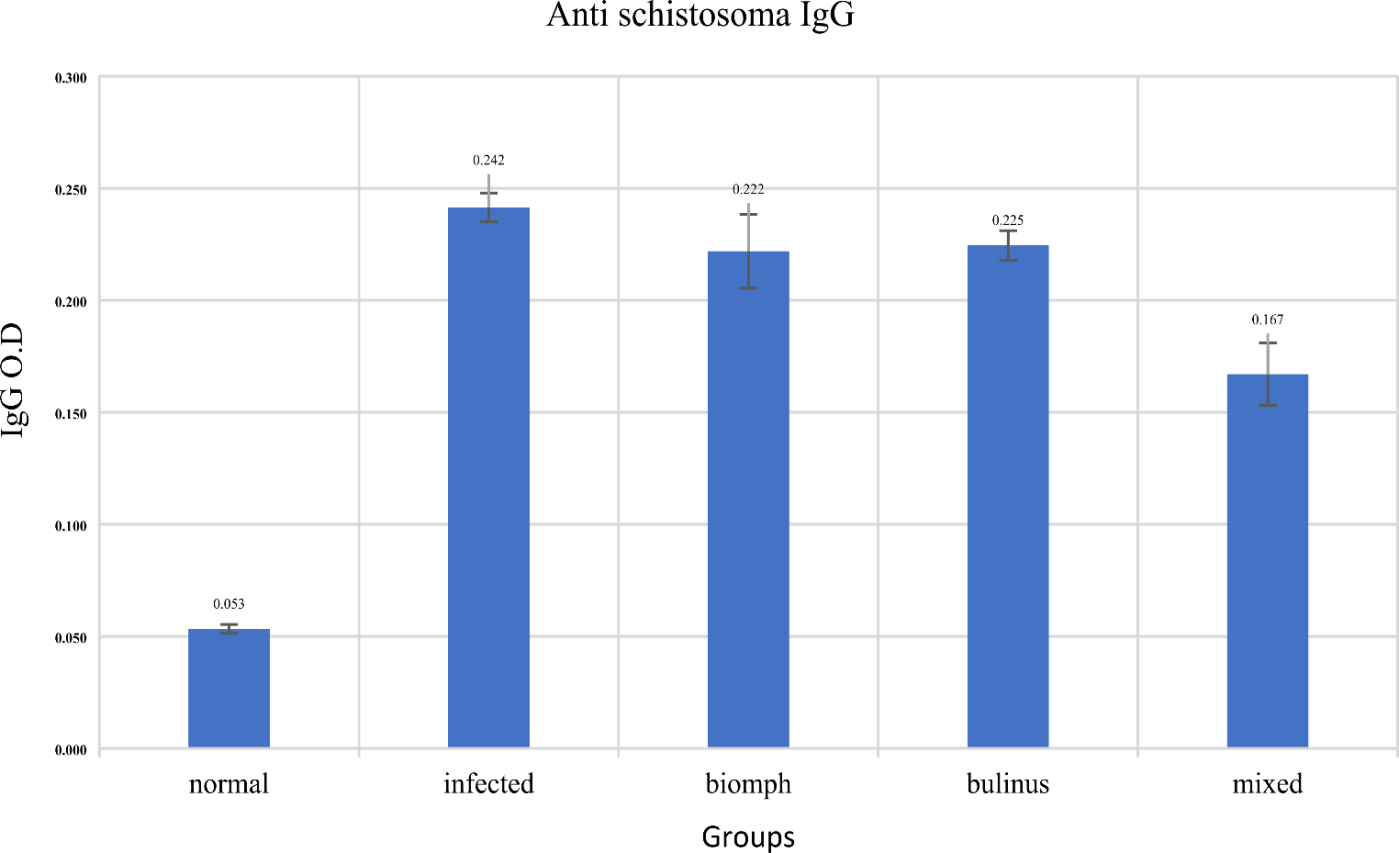
The mean optical density (O.D.) for anti-Schistosoma IgG across different groups

There was a significant increase in O.D. for anti-Schistosoma IgG in the infected and all pre-treatment groups (Biomphalaria, Bulinus and the combination) group compared to the negative control (*p* < 0.001), with no significant difference between the infection and pre-treatment with Biomphalaria and Bulinus groups (*p* > 0.05). The optical density for anti-Schistosoma IgG was significantly higher *P* < 0.01 in mixed group compared to the infected control, (Fig. 2; Table 2).

**Table 2:** The mean optical density (O.D.) for anti-schistosoma IgG across different groups

Groups	Normal	Infected	Biomphlaria	Bulinus	Mixed
**IgG** **(O. D.)**	0.053 ± 0.002	0.242 ± 0.006	0.222 ± 0.016	0.225 ± 0.007	0.167 ± 0.014
**Significance**		***	***, NS	***, NS	***, @@

Data represented as mean ± standard error. ***Significant difference at *p* < 0.001 in comparison to normal, @@ Significant difference at *p* < 0.01 in comparison to infected, NS is non-significant

### Gene Expression Levels

**Fig. 3:**
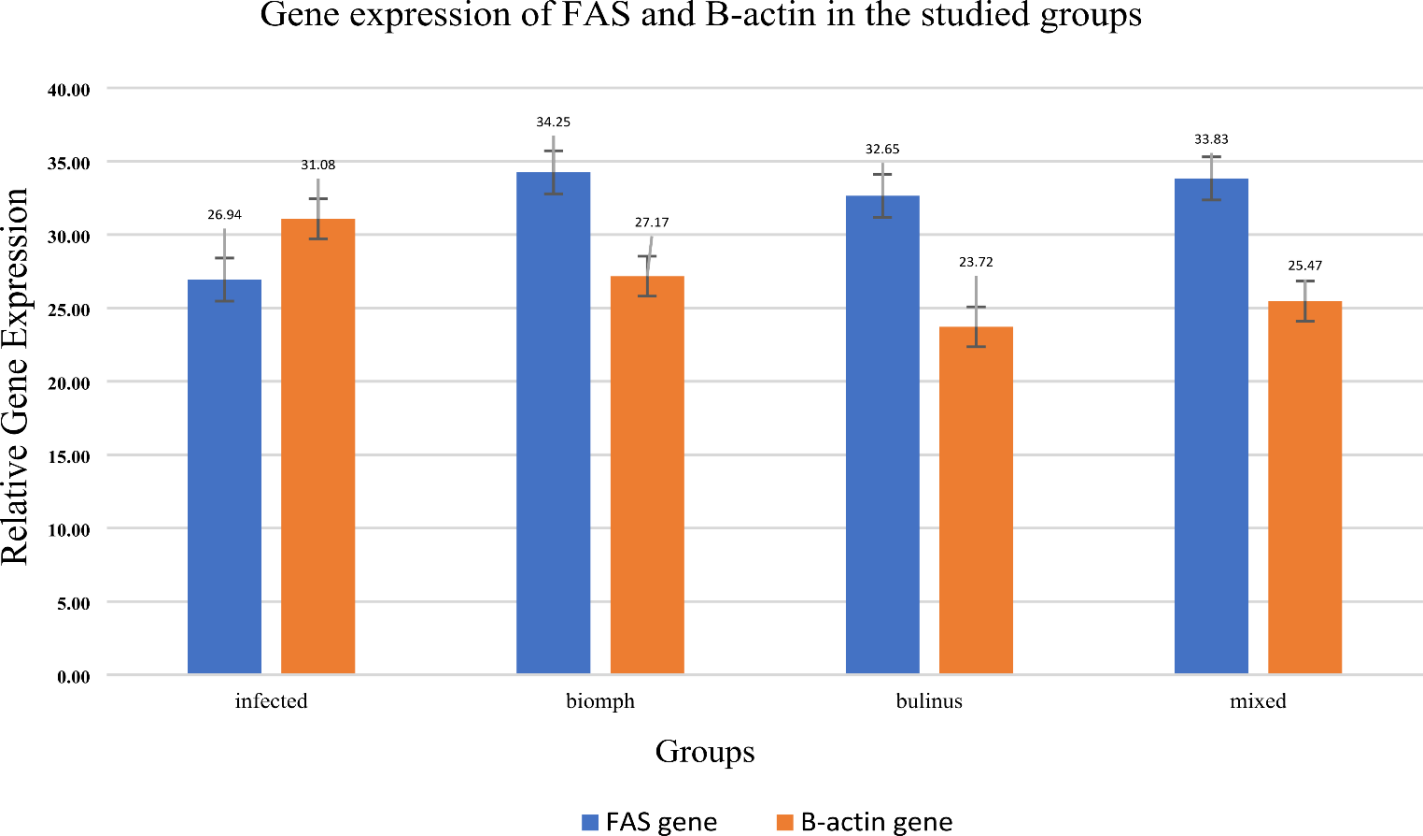
Expression levels of FAS and B-ACTIN genes across treatment groups. Data represented as mean ± standard error. ***Significant difference at *p* < 0.001, ** Significant difference at *p* < 0.01, * Significant difference at *p* < 0.05 in comparison to the infected controls

The analysis of FAS and *β* -actin gene expression across different experimental groups revealed notable differences between treatments. The infected control group exhibited baseline levels of FAS and *β* -actin at 26.94 ± 0.45 and 31.08 ± 1.30, respectively, which served as the reference point for subsequent comparisons. In the *Biomphalaria* group, FAS expression significantly increased to 34.25 ± 2.17 (*p* < 0.05), while *β*-actin expression was notably reduced to 27.17 ± 0.97 (*p* < 0.001) relative to the infected control. Similarly, the *Bulinus* group demonstrated a substantial elevation in FAS levels, reaching 32.65 ± 0.72 (*p* < 0.001), alongside a significant reduction in *β*-actin levels to 23.72 ± 0.98 (*p* < 0.001). The group treated with a *mixed nucleoprotein*, consisting of both *B. alexandrina* and *B. truncatus*, also displayed elevated FAS expression at 33.83 ± 1.24 (*p* < 0.01) and significantly reduced B-Actin expression at 25.47 ± 1.30 (*p* < 0.01) compared to the control (Fig. 3).

## Discussion

Schistosomiasis is an endemic tropical disease Schistosomiasis has been eliminated at many levels by controlling the intermediate host and the primary host by many means including chemical and plant-based [26]. Many authors evaluate many plants extract and synthetic nanoparticles against intermediate host and larval stages of *schistosoma mansoni* [27]. Nucleoproteins (NPs) extracted from *Biomphalaria alexandrina* snails to be used against *schistosoma mansoni* in mice, the effect on *S. mansoni* adult females was improved than adult males at the maximum concentration of 100 µl/ml after 72 h with a 60% lethal effect while 48 h at the same concentration had a healthier lethal impact on males (25%) than (20%) in females [28]. All those results confirmed that, outcome of any treatment vary on many physical parameters sex type, also increasing in concentration and time of exposure [32]. approved that adult female worms were more susceptible than males after in vitro incubation with cnicin especially at low concentrations while, no death in male worms was observed. Maybe that is dependent on the pharmacological possessions of the drugs used. On the conflicting, [7] stated that the ratio of worm reduction was significantly increased in *S. mansoni* infected mice in collective treated groups with PZQ and omeperazole at 6 weeks post infection as associated to mice treated with PZQ or omeprazole alone.

This study investigated the effects of proteins derived from the intermediate hosts *Biomphalaria* and *Bulinus* on the immune response and parasitology in mice infected with *Schistosoma mansoni*. The results demonstrate significant reductions in worm burden, increased anti-Schistosoma IgG levels, and notable changes in gene expression, supporting the hypothesis that these snail-derived proteins can induce a protective immune response against schistosomiasis.

Mice administered with either *B. alexandrina* or *B. truncatus* experienced a significant dip in the worm burden, achieving over 68% reduction in worm counts compared to the control group. These findings are consistent with previous studies [11, 39], which proposed that proteins from *B. alexandrina* can provoke and enhance the resistance to schistosomiasis [39]. also, demonstrated that specific proteins from *Biomphalaria glabrata* can activate immune responses in mice, leading to reduced parasitic loads. Alongside [3], illustrated the immunomodulatory effects of *B. truncatus* proteins that possibly can impact the host immune pathways to antagonize the pathogenesis of schistosomiasis.

Surprisingly, the worm load recovered from mice administered with the mixed nucleoproteins showed less reduction by 44.25%, indicating that the combined proteins may not yield additive protective effects. Yet, this unexpected effect promotes a further investigation into the interactions between different protein types and their collective impact on immunity, as some combinations may lead to antagonistic effects that diminish overall efficacy [21].

The significant surge in anti-Schistosoma IgG levels across all experimental groups indicates a robust humoral immune response compared to the controls. These elevated IgG levels may verify the potential effects of nucleoproteins in stimulating B cell activation, leading to enhanced antibody production against schistosome antigens. Interestingly, the optical density measurements that showed no significant differences among the various nucleoproteins-treated groups may indicate that the immune system attains a saturation point in response to the proteins. This finding might be supported by a previous research study [6], which recorded the high antibody responses through various strategies involving *Biomphalaria* derivatives [24].

The analysis of FAS and β-Actin gene expressions reveals important insights into the mechanisms of immune modulation. The upregulation of FAS/R gene expressions in the *B. alexandrina- and B. truncatus-treated* mice suggests that these proteins may promote apoptosis via the FAS/R ligand pathway in infected cells and potentially lead to the clearance of the parasite as illustrated in previous studies [19]; [30]; Wajant [38], 

The downregulation of β-Actin across nucleoproteins-treated mice suggests a shift in cellular metabolism, possibly indicating a reprogramming of immune cells to prioritise anti-parasitic functions over general cellular processes. This modulation of β-Actin expression is particularly noteworthy, as it implies a significant alteration in the cytoskeletal dynamics of immune cells, which are critical for their migration and function during an immune response [22]. It is worth mentioning that the increase of FAS gene expressions concomitant with the decrease of β-actin levels across all treated groups underscores the potential modulatory effect of these interventions on gene expressions during *S. mansoni* infection.

The findings of this study may significantly contribute to a growing body of literature [13, 23], highlighting the potential of applying snail proteins in immunization strategies against schistosomiasis.

## Conclusion

In a nutshell, this study featured the promise of utilizing snail-derived proteins to induce tolerance to murine schistosomiasis *mansoni*. The significant reductions in worm burden, enhanced IgG levels, and favorable gene expression profiles indicate that these proteins could form the basis of novel immunotherapeutic strategies. Future research should focus on elucidating the specific components responsible for these effects and optimizing combinations for maximal protective efficacy against schistosomiasis.

## Electronic Supplementary Material

Below is the link to the electronic supplementary material.

Supplementary Material 1

## Data Availability

No datasets were generated or analysed during the current study.
