# Supplementary figures and images for: Approaches of Inducing Tolerance to Murine Schistosomiasis mansoni applying Biomphalaria and Bulinus Proteins
Source: Acta Parasitol. 2025 Jan 24;70(1):41. doi: 10.1007/s11686-025-00988-2 (PMC11761983; doi:10.1007/s11686-025-00988-2)

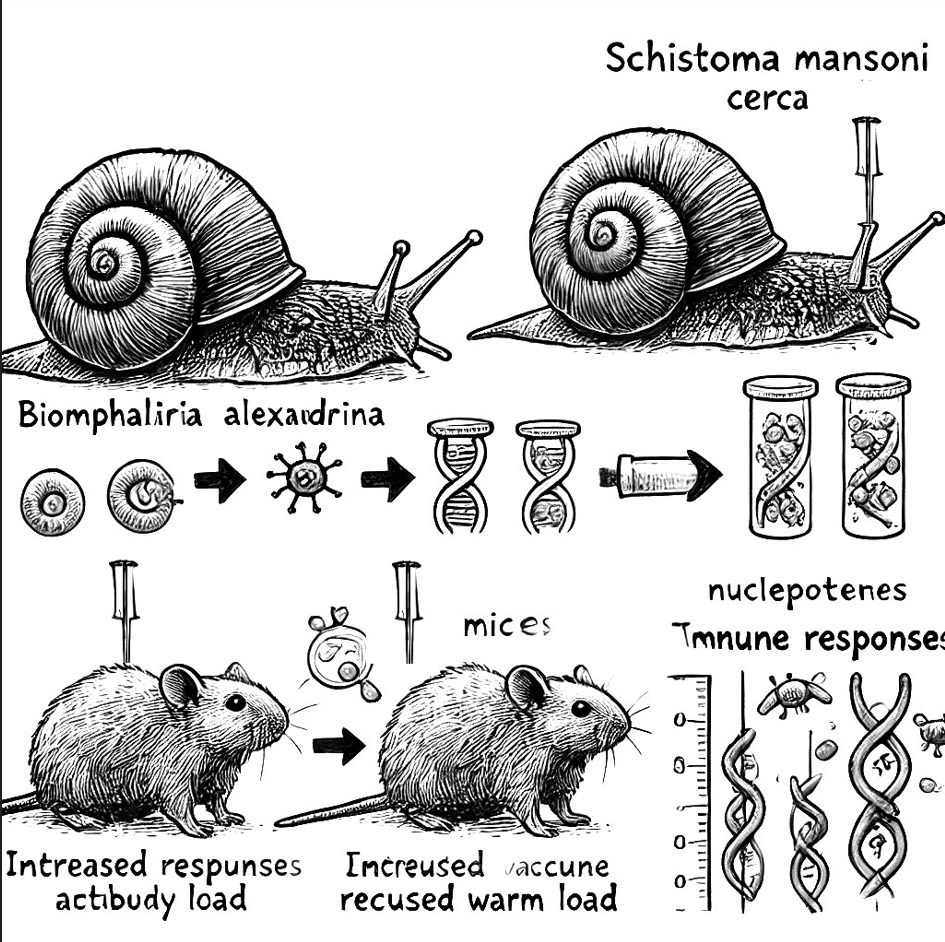

Supplement: Supplementary file 1 — Supplementary Material 1 [file 11686_2025_988_MOESM1_ESM.jpg]
